# A Case of Thrombotic Thrombocytopenic Purpura and ST-Elevation Myocardial Infarction: An Unusual Correlation

**DOI:** 10.7759/cureus.36039

**Published:** 2023-03-12

**Authors:** Adekunle E Omole, Azka Ali, Kayode E Ogunniyi, Danish Waqar, Opeyemi Tobalesi, Omar Rahim, Ayoola Awosika

**Affiliations:** 1 Anatomical Sciences, American University of Antigua, College of Medicine, Saint John, ATG; 2 Internal Medicine, Chicago Medical School/Rosalind Franklin University of Medicine and Sciences, North Chicago, USA; 3 Internal Medicine, University Hospital of North Durham, Durham, GBR; 4 Internal Medicine/Nephrology, Loyola University Medical Center, Chicago, USA; 5 Internal Medicine, College of Health Sciences, University of Ilorin, Ilorin, NGA; 6 Internal Medicine, Naseer Teaching Hospital, Peshawar, PAK; 7 College of Medicine, University of Illinois, Chicago, USA; 8 College of Health Sciences and Professions, Ohio University, Athens, USA

**Keywords:** acute thrombotic microangiopathy, acquired ttp, ttp, thrombotic thrombocytopenic purpura, acute coronary syndrome, myocardial infarction

## Abstract

Thrombotic thrombocytopenic purpura (TTP) is a rare and potentially devastating blood disorder depicted by thrombocytopenia, fever, widespread small vessel hemolytic anemia, and neurological symptoms. The involvement of the renal and neurological systems is frequently reported in TTP; however, TTP-induced acute coronary syndrome is not widely reported. We describe a case of myocardial infarction induced by TTP in a patient who presented with the typical manifestation of acute coronary syndrome. Echocardiogram revealed a myocardial injury, and detailed investigations revealed increased levels of troponin I, lactate dehydrogenase, diminished levels of haptoglobin and von Willebrand factor-cleaving protease, and schistocytes on peripheral smear, suggestive of TTP-induced myocardial infarction. His condition was stabilized after commencing plasmapheresis, steroids, and rituximab. The initial steps in the management of this patient involve the prompt administration of steroids and the urgent start of plasmapheresis to increase platelet count.

## Introduction

Thrombotic thrombocytopenic purpura (TTP) is a subset of thrombotic microangiopathies manifesting low platelet count and widespread microangiopathic hemolytic anemia. TTP commonly affects the renal and neurological systems [1]. TTP results in disseminated microthrombi in small blood vessels and is associated with diminished titers of von Willebrand factor cleaving protease, a disintegrin-like, and metalloproteinase with a thrombospondin type 1 repeat motif, member 13 (ADAMTS-13) [2]. TTP can be hereditary or acquired based on a genetic deficiency of ADAMTS-13 due to gene mutations or anti-ADAMTS-13 autoantibodies, respectively. Involvement of the kidney and neurological system is widely described in the literature; however, heart involvement in TTP is uncommon [3]. However, cardiac involvement is frequently seen during a post-mortem examination [3]. Herein, we present a patient who manifested with TTP and ST-segment elevation myocardial infarction (STEMI) as an initial manifestation of the TTP.

## Case presentation

A 56-year-old male with a known case of diabetes mellitus and hypertension was brought to the emergency department with chest pain for the last eighteen hours. The pain was sudden in onset, progressive, and radiating to the neck. The chest pain is worse on exertion and minimal activity. No aggravating and relieving factors are associated with dyspnea on exertion. He also reported intermittent headaches and blood in the urine for the last twenty days. He had no history of bleeding disorders or any family history of similar diseases with no allergies. He was compliant with his medications. He denied polyuria, dysuria, nausea, vomiting, abdominal pain, or burning micturition.

**Figure 1:**
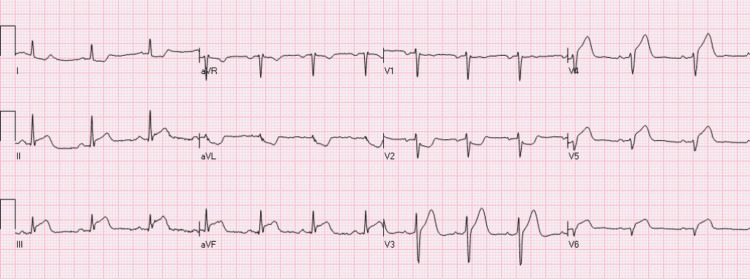
Electrocardiograph demonstrating ST-segment elevation in leads 2, 3, and aVF with reciprocal changes in the aVL lead aVF- Augmented vector Foot; aVL- Augmented vector Left

On examination, he was irritable, hemodynamically stable, and well-oriented in time, place, and person and had mild icterus with no lymphadenopathy and any visceral enlargement. An urgent electrocardiogram (EKG) revealed ST-segment elevation in inferior leads with reciprocal changes in aVL lead (Figure 1). Initial laboratory investigations were significant for thrombocytopenia and anemia with high serum creatinine levels (Table 1). Evidence of myocardial injury was observed due to elevated cardiac enzymes and troponin I level. A provisional diagnosis of inferior wall STEMI was made; however, no antiplatelet medicine and coronary intervention were initiated because of severe thrombocytopenia and hematuria.

**Table 1:** Initial results of hematological investigations MCV: mean corpuscular volume

Parameter	Lab value	Reference range
Hemoglobin	9.8 g/dL	13.5-16.5
Red cell count	3.9 million cells/uL	4.7-6.1
Hematocrit	33%	41-53
MCV	81.2 fl	80-100
Platelet count	17000/uL	150,000-350,000
White cell count	10500/uL	4000-11000
Blood urea nitrogen	37 mg/dL	08-20
Serum creatinine	2.5 mg/dL	0.4-1.3

Further investigations revealed elevated lactate dehydrogenase (LDH), low haptoglobin, and elevated serum lipase levels with many schistocytes on the peripheral blood smear, leading to a high suspicion for TTP. A confirmatory test for the serum level of ADAMTS-13 was low, coupled with an elevated ADAMTS inhibitor titer, thus confirming our diagnosis of acquired TTP (Table 2). He underwent transthoracic echocardiography, which revealed mild to moderate left ventricular systolic dysfunction with an ejection fraction of 40-45% and moderate anteroseptal and inferoseptal hypokinesia.

**Table 2:** The results of biochemical parameters CKMB: creatine kinase-myocardial band, ADMATS-13: a disintegrin-like and metalloproteinase with thrombospondin type 1 motif, member 13

Parameter	Lab value	Reference range
CKMB	74 IU/L	5-25
Lactate dehydrogenase	1151 IU/L	90-192
Troponin I	1.1 ng/dL	< 0.05
Haptoglobin	21 mg/dL	34-200
Total bilirubin	2.1 mg/dL	0.3-1.2
Serum lipase	548 IU/L	0-160
ADAMTS-13	14%	< 66%
ADAMTS-13 antibody titer	129 IU/ml	< 12

**Figure 2:**
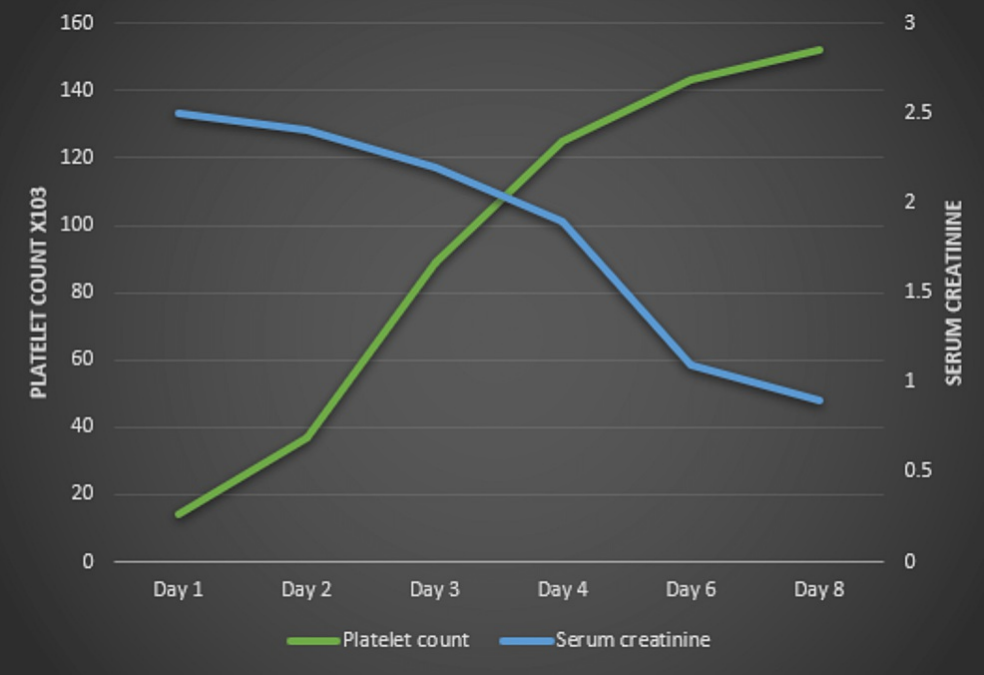
Trend of platelet count and serum creatinine during the hospital stay

He was commenced on plasmapheresis and was monitored closely. He underwent plasmapheresis daily and intravenous methylprednisolone followed by weekly rituximab at 375 mg/m^2^ body surface area. His symptoms started improving over the subsequent days, a remarkable increase in platelet count was observed, and a declining trend in serum creatinine was noted on admission day six (Figure 2). He was managed conservatively with clopidogrel, atorvastatin, and metoprolol. He remained in the hospital for 15 days, and the plasmapheresis session was tapered after two doses of rituximab. He was discharged with a regular follow-up with his physician and referred to the cardiologist for further management.

## Discussion

Although cardiac involvement is a known complication of TTP, chest pain with evidence of ST-segment elevation myocardial infarction (STEMI) as the initial symptom is rarely seen in the literature. TTP-induced cardiovascular manifestations may vary from asymptomatic (raised cardiac biomarkers) to sudden cardiac death, myocarditis, arrhythmias, heart failure, or myocardial infarction [4,5]. An acute coronary syndrome due to TTP may result from the formation of microthrombi in the myocardial microvessels making it challenging to differentiate from acute coronary syndrome due to atherosclerosis [6]. The exact prevalence of cardiovascular involvement in TTP is unknown; however: a recent review article of 56 patients diagnosed with TTP showed platelet-rich microthrombi and extensive cardiac involvement despite the rarity of symptoms on autopsy [7]. A recent study on the prevalence of cardiac involvement in TTP reported that 14 out of 32 patients had myocardial infarction [8].

Early diagnosis and management are mandatory to prevent high mortality from TTP-induced cardiac morbidity. Severe morbidity and mortality are reported to be higher in TTP patients with high levels of positive cardiac biomarkers [9]. It has been reported that the mortality risk increases threefold in patients with TTP who have serum troponin levels greater than 0.25 ug/ml [10]. Managing myocardial infarction in patients with TTP can be difficult due to low platelet count, which makes it rather difficult to start dual antiplatelet therapy, including aspirin and clopidogrel, as antiplatelet medicine further augments the bleeding risk in TTP. Although giving aspirin has been found to inhibit platelet aggregation and decrease the death rate in TTP patients, adding clopidogrel to the treatment regimen is contentious since it can cause TTP itself [11]. A recent study reported a reduction in mortality at day 15 after adding ticlopidine to plasmapheresis. Additionally, aspirin can be given as an adjuvant therapy when the platelet count is above 50,000, with no risk of bleeding [12].

MI management in TTP is tricky because of severe thrombocytopenia and acute kidney failure, which usually impedes dual antiplatelet therapy, cardiac catheterization, or percutaneous coronary intervention [7,13]. Standard recommendations advocate a complete cardiac workup with clinical examination, EKG, echocardiography, and serum assessment of cardiac enzymes. In TTP patients with myocardial injury, immediate plasmapheresis is mandatory to prevent further cardiac injury and mortality [3,14]. Additionally, continuous cardiac monitoring is essential in these patients, given an increased risk of fatal arrhythmias.

In our case, the patient presented with STEMI, characterized by the typical presentation of acute coronary syndrome and ischemic changes on EKG. Severe thrombocytopenia elevated LDH and haptoglobin, and schistocytes on peripheral blood smear led to the diagnosis of TTP. A robust causal relationship between TTP and non-ST-segment elevation myocardial infarction (NSTEMI) is challenging to establish in the same patient. Our patient underwent urgent plasmapheresis, steroid administration, rituximab, and conservative treatment with clopidogrel, atorvastatin, and metoprolol which significantly improved his clinical condition.

## Conclusions

TTP-induced myocardial infarction is a rare but potentially serious medical condition. Given the high morbidity and mortality of TTP-induced cardiac injury, all patients with TTP undergo routine evaluation with EKG, cardiac enzymes, and telemetry. Thus, early initiation of the cornerstone of TTP treatment, including plasma exchange therapy and steroid administration, should be instituted promptly.
